# Why a renewed focus on regional governance is needed post-2015

**DOI:** 10.1177/1468018115600123e

**Published:** 2015-12

**Authors:** Erica Penfold

**Affiliations:** The South African Institute of International Affairs South Africa

The **68th session of the World Health Assembly** in May 2015 took as its focus ‘Ebola: Ending the current outbreak, strengthening global preparedness and ensuring the WHO’s capacity to prepare for and respond to future large-scale outbreaks and emergencies with health consequences’, and ‘Health in the post-2015 development agenda’.

What was missing from the agenda was whether and how regional health governance in Africa – as in other continents around the world – can be strengthened to enable the WHO and other actors in the field of health to prepare for and respond to future large-scale outbreaks of communicable diseases. This challenge is closely related to that of mainstreaming health in development agendas, something which the post-2015/Sustainable Development Goal agenda has not yet adequately grasped. The approach to health remains in the hands of more developed nations who determine developing nation agendas. Put simply, investing in robust, comprehensive health systems is a vital social as well as an economic investment. As the Ebola outbreak showed, the damage to West African economies was a staggering half a billion dollars in 2014, which is nearly 5% of the three affected countries GDP. This will take years to recover from (The World Bank, 2015).

The argument for strengthening regional health governance is evident in the work achieved by the Union of South American Nations and the WHO’s South East Asian Regional Office. Regional health governance in these two regions focuses very much on collaborative governance between states on key health issues, including HIV/AIDS, Chagas, malaria and regional support for regulation of medicines and development of healthcare systems in both regions, which is seen to be relatively effective, despite many administrative and political challenges.

The situation in sub-Saharan African is however quite different. Considering the size of the continent, the proliferation of disease and poor established healthcare infrastructure, the path of communicable diseases is more deadly and difficult to control. This is evidenced by the Ebola outbreak in 2014 and the proliferation of HIV/AIDS on the continent over the last two decades. Regional organisations in West, East and Southern Africa, despite having long established presences, are heavily reliant on the intervention of donors in assisting to curb the spread of disease, develop adequate healthcare systems and horizontal approaches to tackling communicable and NCDs. The regional successes are underplayed by the role that donors play and the involvement of the WHO regional bodies, multilaterals and major organisations including the Bill and Melinda Gates Foundation and the Clinton Health Access Initiative.

The reason for this could be a different understanding of regionalism and regional health governance in sub-Saharan Africa. The role of regional organisations differs from that of their South American and South East Asian equivalents. The focus of regionalism in sub-Saharan Africa is very much on regional integration, economic development and the creation of monetary unions to promote economic improvement. As such, the dedicated health arms are not developed as regional health governance bodies.

The SADC has a dedicated policy directorate for social development in general but there is no specific regional health body that is concerned with regional governance and the control of communicable and NCDs. Notwithstanding, the SADC Secretariat has had successes in developing cross-border health initiatives for malaria and HIV/AIDS. Although the focus of controlling disease is at a national level, in the SADC Member States, there is a means of regional involvement, although not direction. The SADC Secretariat plays the role of facilitator for regional programmes and sometimes enforces its role as approval mechanism for regional plans developed by national and donor entities, but does not play an active role in implementation or monitoring and evaluation.

The focus at the WHA this year was still very much a vertical approach to controlling disease and support for development goals. But without concerted action to develop robust comprehensive health systems whereby health systems are supported and developed by national governments, with correct facilities and trained staff, the disease-driven approach will be less than effective in the long term. Donors need to shift their thinking to these realities and the challenges of leadership in these regions. Donors can determine the development agenda to an extent but if plans are not approved by regional bodies or governments, there is very little programmes can do to continue with implementation until strategies are approved. Donors often have to find ways to bypass the rule of organisations in order to continue with the work they are doing. As with the Ebola outbreak in West Africa, regional leadership in providing healthcare to the population has been called into question. The absence of leadership could be seen as a causal factor for the continent’s health challenges. Dr Margaret Mungherera, past president of the World Medical Association writes, ‘Developing leadership capacity should therefore be the main emphasis of any eff ort aiming to reduce Africa’s disease burden’ (Ezeh, 2015;).

In this context, a beneficial addition to this discussion could be whether renewed focus on the creation of regional health governance bodies, or that sub-Saharan regions take the lead in developing healthcare on a regional scale, as opposed to a national–regional bottom-up approach, has the potential to benefit disease control in each region.

The imbalance of political power in sub-Saharan Africa between governments, civil society and donors and the proliferation of undemocratic governments who do not always engage with civil society is also perhaps a dominant factor in hindering the growth of strengthened regional (health) governance in the continent. Additionally, the ambiguous role played by the African Union (AU), and its positioning against SADC, ECOWAS and the EAC, creates confusion as to whether the AU is a regional body or continental body responsible for coordinating regional health response collaboratively with each regional organisation. The weight of power carried by countries such as Nigeria, South Africa and Kenya often creates a regional imbalance as these countries often carry the weight of donor funding in each region, or can create problems in how policy is developed.

As the highest decision-making body for global and regional health, the WHO WHA needs to consider elevating the status of regional health organisations in Sub-Saharan Africa and consider providing increased technical assistance for strengthened horizontal healthcare systems. It also needs to provide expertise for the region [continent?] to enable regional health bodies to manage communicable disease outbreaks and provide support for NCDs. This would address both the key themes of the Assembly while also the priorities of the SDGs.

Key resolutions made which encourage regional collaboration included the resolution to improve access to sustainable supplies of affordable vaccines. This is an imperative step for low- and middle-income countries needing to immunise their populations. The resolution requests that the WHO coordinate efforts to address progress for the Global Vaccine Action Plan, endorsed by the WHA in 2012. Member states are urged to increase vaccine pricing transparency and ‘explore’ pooling the procurement of vaccines. This relates to agreements made for the SADC Pharmaceutical Business Plan, which expired in 2013 and as yet has not been renewed. The SADC Secretariat has not ensured that vaccine procurement is a priority in the region, but perhaps with the adoption of the WHA resolution for vaccines, this may become a renewed and urgent priority in the region. There should also be consideration of integration with the WHO on regional governance of vaccines.

Considering Ebola’s continued prevalence in West Africa, and the continued threats it poses to health security and justice throughout the continent, and beyond, together with the need for a renewed focus on health in the post-2015 development agenda, thinking and acting regionally is a vital cornerstone to efforts to reintegrate and redevelop health systems on the continent. It is time to rethink the ways in which global and national actors engage with regional integration and governance. This must be a priority for the 2016 meetings (and beyond) of the assembly as it must too in time for the implementation phase of the SDGs.
